# Exploring the role of shame and self-compassion on the link between fibromyalgia symptoms and depression: Insights from mediation and moderation analyses

**DOI:** 10.1177/13591053251331286

**Published:** 2025-04-15

**Authors:** Judite Fortuna, Ana M Pinto, José A. P. da Silva, Rinie Geenen, Paula Castilho

**Affiliations:** 1 Faculty of Psychology and Educational Sciences, University of Coimbra, Portugal; 2 Faculty of Psychology and Educational Sciences, Centre for Research in Neuropsychology and Cognitive and Behavioural Intervention, University of Coimbra, Portugal; 3 Faculty of Medicine, Coimbra Institute for Clinical and Biomedical Research (i.CBR), University of Coimbra, Portugal; 4 Coimbra Hospital and University Centre, Portugal; 5 Utrecht University, The Netherlands; 6 Altrecht Psychosomatic Medicine Eikenboom, The Netherlands

**Keywords:** fibromyalgia, depressive symptoms, shame, compassionate self-responding, mediation/moderation

## Abstract

The intricacies of the fibromyalgia-depression link accentuate the need to further explore underlying psychosocial mechanisms. External shame resulting from fibromyalgia’s nature and associated impairment may increase the risk for depression. We explored whether being supportive and compassionate toward one’s perceived shortcomings would potentially weaken this association. This cross-sectional study comprised 138 women with fibromyalgia. Participants were recruited via patient associations and invited to complete an online survey. Descriptive, correlational, mediation and moderation analyses were performed to test the driving hypotheses. Both mediation and moderation analyses accounted for approximately 40% of the variance in depressive symptoms. Fibromyalgia severity was directly and indirectly— through external shame— associated with depressive symptoms. The shame-depressive symptoms link was weaker in participants with greater self-compassion skills. Findings point to the importance of shame and self-compassion and the need to address them in research and clinical contexts.

## Introduction

Fibromyalgia is a chronic and multidetermined condition that affects around 1.8% of the general adult population worldwide (Heidari et al., 2017) and greatly impacts individual and social functioning (Arnold et al., 2008; Galvez-Sánchez et al., 2019). Characterized by a set of symptoms such as widespread pain, fatigue, sleep impairment, and cognitive dysfunction (Wolfe et al., 2011; Wolfe et al., 2016), fibromyalgia is a challenging condition for both patients and clinicians.

Many people with fibromyalgia have other physical and mental disorders (Pinto, Luís, et al., 2023), which adds to the existing burden, disability, and suffering individuals have to endure (Häuser et al., 2015). Depression is the most prevalent psychiatric comorbidity in fibromyalgia, with pooled point-prevalence estimates between 25% and 45% (Häuser et al., 2015; Kleykamp et al., 2021) and a mean pooled lifetime prevalence of 65% (Løge-Hagen et al., 2019). Fibromyalgia and depression show a bidirectional relationship (Chang et al., 2015). When comorbid, they are associated with more severe phenotypes, worse physical, mental, and quality-of-life outcomes, and poorer treatment adherence and responsiveness (Alciati et al., 2012; Alok et al., 2011; Lange and Petermann, 2010). The pathways contributing to the association between fibromyalgia and depression are multiple and complex, spanning from shared genetic influences and biobehavioral mechanisms to dysfunctional cognitive-affective processes (Geenen et al., 2012; Staud, 2015).

The observation that not all patients with fibromyalgia experience depression underscores the need for a better understanding of potential risk and protective factors (Okifuji et al., 2000). One potential risk factor that has received little attention in fibromyalgia is shame, in spite of research reporting that fear of negative evaluation, shame, and shame-related experiences are prevalent phenomena among people with chronic pain (Asbring and Närvänen, 2002; Nicola et al., 2021; Turner-Cobb et al., 2015).

Shame is rooted in the threat-defense system (Gilbert, 2010)—responsible for tracking down and managing perceived threats—and it consists of an evolutionary response to any form of menace toward the “social self” (Gilbert, 2007). According to the social rank theory, one’s prospect of survival and thriving depends highly on one’s ability to compete for and garner social desirability, dominance, and status (Gilbert, 2000; Stevens and Price, 2000). Fibromyalgia symptoms and associated disability may jeopardize one’s social place by giving rise to a global sense of being disadvantaged or undesirable in relation to other people, a view reinforced by unfavorable social comparison processes (Cabrera-Perona et al., 2017). Fibromyalgia symptoms are, for many patients, pervasive, distressing, and the source of marked functional impairment, disrupting daily living activities, thwarting valued-based goals, and fueling a sense of inadequacy, burdensomeness, shame, and guilt (Arnold et al., 2008; Lempp et al., 2009).

The uncontrollable, unpredictable, and invisible nature of fibromyalgia symptoms, which have no objective biomarker, renders the individual vulnerable to other’s invalidation, criticism, and stigma (Boyington et al., 2015; Mengshoel et al., 2018; Quintner, 2020; Van Alboom et al., 2021) and may contribute to social withdrawal, compromising one’s ability for social connection (Arnold et al., 2008; Cunningham and Jillings, 2006). These negative interpersonal experiences, especially when occurring within the family realm, may contribute to feelings of isolation, worthlessness, a negative internal dialog (Arnold et al., 2008; Boring et al., 2021; Lempp et al., 2009), and even social pain (Ghavidel-Parsa and Bidari, 2021).

Fibromyalgia and pain may also impact the representation of one’s body (Galvez-Sánchez et al., 2019; Kim et al., 2012), which mismatches the socio-culturally valued “healthy body” (Thompson and Kent, 2001) and, therefore, comes to be perceived as unfitted, defective, or lesser (Gilbert, 2017). Given that bodies constitute “extensions of ‘who we are’ ” (p. 3), these negative judgments may generalize over time (Gilbert, 2017). Coupled with fibromyalgia symptoms, disability, and associated shaming experiences, this can become central and profoundly change how one sees oneself—a phenomenon that has been described as a “pain assault to the self” (Asbring, 2001; Galvez-Sánchez et al., 2019; Paschali et al., 2021). Shame and depression may emerge as responses to this perception of involuntary social devaluation and the ensuing fear of being attacked or rejected by others and of losing valued resources (Gilbert, 2000; Stevens and Price, 2000).

Shame has external and internal aspects referring to the source of unfavorable evaluations, that is, how one believes to be perceived by others versus how one perceives oneself (Gilbert, 2007). Shame typically involves negative social and self-related judgments (e.g. of one as inferior and worthless and others as critical and rejecting), negative affect, and defensive actions (e.g. concealment, positive self-presentation). Despite its survival value, when extreme and persistent, shame is associated with a wide range of maladaptive processes (Castilho et al., 2017; Gilbert and Miles, 2000)—some of which are recognized precursors of depression such as entrapment and defeat (Gilbert et al., 2002; Taylor et al., 2011)—and with psychopathological and somatic symptoms (Cândea and Szentagotai-Tătar, 2018; Kealy et al., 2018; Kim et al., 2011; Nechita et al., 2021; Pineles et al., 2006). Shame may increase one’s vulnerability to disease by restraining access to positive socio-emotional resources (Cacioppo and Patrick, 2008), compromising help-seeking and disclosure, and negatively shaping the therapeutic alliance and influencing treatment adherence (Gilbert, 2017).

Crucial in the understanding of (mental) health and resilience (MacBeth and Gumley, 2012; Phillips and Hine, 2021), self-compassion has been proposed to counteract the pernicious impact of shame, to constitute a positive resource to cope with personal shortcomings and adversity such as that imposed by challenging medical conditions and to act as a buffer in the development of depressive symptoms (Kim et al., 2011; Lanzaro et al., 2021). While a variety of flows and definitions have been suggested (Strauss et al., 2016), self-compassion can be conceptualized as an attunement with one’s suffering, acknowledged as a universally shared phenomenon and approached with openness and kindness, together with a motivation to act and ease it (Gilbert, 2005; Neff, 2003). Self-compassion is rooted in the soothing and affiliative system, a system associated with a caregiving mentality and responsible for one’s ability to self-regulate (Gilbert, 2020; Seppälä et al., 2017).

Research has shown that self-compassion: (a) may dampen the impact of pain-related disability on depression (Carvalho, Trindade, et al., 2020); (b) is associated with a reduced perceived disease impact and associated psychological distress (Costa and Pinto-Gouveia, 2013; Hughes et al., 2021); (c) is associated with better coping and physiological and cognitive-affective regulation (Lanzaro et al., 2021; Maratos and Sheffield, 2020; Sirois et al., 2015); and (d) may act as a mediator in the relationship between beliefs about future changeability—a key variable related to entrapment and behavioral change (Hellström et al., 2000)—and pain severity (Chang et al., 2019). On the other side of the continuum is uncompassionate self-responding (Neff, 2023), which has been related to psychological inflexibility-related processes and to depressive symptoms both cross-sectional and longitudinally (Carvalho, Pinto-Gouveia, et al., 2020; Carvalho, Trindade, et al., 2020). Noteworthy, self-compassion was found to be lower among people with fibromyalgia when compared to other rheumatic populations such as ankylosing spondylitis and rheumatoid arthritis (Sirois and Hirsch, 2019).

The potential protective role of self-compassion is further supported by compassion-focused interventions, which have shown promising results in improving pain-related outcomes, psychological distress, a more adaptive self-to-self relationship and coping styles, and increased well-being across different non-clinical and clinical samples (Kirby et al., 2017; Leaviss and Uttley, 2015), including people with chronic pain and other chronic physical illnesses (Austin et al., 2021; Kılıç et al., 2021; Penlington, 2019). Despite the sparking interest for self-compassion in chronic pain research, its potential role and interrelationship with other variables remains underexplored in fibromyalgia.

The twofold aims of the present study in people with fibromyalgia was were to get insight into (1) the potential relevance of external shame as a threat fostering depressive symptoms and (2) the health-protective role of self-compassion in case of external shame. To that aim, we postulated simple mediation and moderation models including fibromyalgia symptom severity as an independent variable, depressive symptoms as the dependent variable, external shame as a mediator, and self-compassion as a moderator of the shame-depression link. Our hypotheses were: (1) fibromyalgia severity is significantly associated with depressive symptoms both directly and indirectly through external shame; and (2) the association between external shame and depressive symptoms varies as a function of compassionate self-responding levels, with greater self-compassion being associated with a weaker association between shame and depressive symptoms.

## Methods

### Participants and procedures

This was a cross-sectional study conducted on a sample of 138 women with fibromyalgia. Participants were recruited online through two patients’ associations and an online institute for people with fibromyalgia using non-probability convenience sampling. Inclusion criteria comprised an established diagnosis of fibromyalgia (self-report), being aged between 18 and 65 years, and being able to read and write Portuguese. Information about the study’s objectives, eligibility criteria, and voluntary nature of participation was provided to participants. Data confidentiality and anonymity were guaranteed. Informed consent was obtained from all participants before entering the study. The assessment protocol was administered through the online tool LimeSurvey® and took approximately 30 minutes to complete. Participants did not receive any compensation for their participation in the study. The present study was approved by the Ethics Committee of the University of Coimbra, Coimbra, Portugal, and followed all ethical requirements.

### Instruments

The online questionnaire included sociodemographic and clinical data comprising age, education, marital status, working status, socioeconomic level, age of first symptoms, age of diagnosis, who performed the diagnosis, comorbidities, and current medication intake. Four self-report questionnaires were included.

*Other As Shamer Scale 2* (OAS2; Matos et al., 2015) a shorter version of the OAS (Goss et al., 1994), this scale is designed to measure one’s perception of existing negatively in the mind of others. It comprises eight items rated using a 5-point Likert scale (0 = “never” to 4 = “almost always”), with higher scores indicating greater levels of external shame. Examples of items include: ‘*Other people see me as small and insignificant*’, and ‘*Other people see me as not measuring up to them.*’ The OAS2 showed strong correlations with the extended version of the scale and good psychometric properties, including good internal consistency (α = 0.82). The scale’s internal consistency in this study was excellent (α = 0.94).

*The Self-Compassion Scale – Short Form* (SCS-SF; Castilho et al., 2015; Raes et al., 2011) is a brief version of the SCS (Neff, 2003), used to measure the quality of relating kindly and compassionately to oneself. This instrument comprises 12 items rated on a 5-point Likert scale (1 = “never”; 5 = “always”). While different factor solutions have been proposed, we decided to use the two-factor solution, encompassing the factors “critical self” and “compassionate self” (Kotera and Sheffield, 2020; Muris and Petrocchi, 2017). The “compassionate self-responding” subscale includes items such as “*When something upsets me I try to keep my emotions in balance*,” and “*I try to see my failings as part of the human condition*.” On the other hand, the items composing the “uncompassionate self-responding subscale” include “*I’m disapproving and judgmental about my own flaws and inadequacies*” and “*When I fail at something that’s important to me, I tend to feel alone in my failure*.” For the purpose of this study, only the “compassionate self-responding” factor was used, for which an excellent internal consistency (α = 0.91) was found.

*Fibromyalgia Impact Questionnaire-Revised* (FIQR; Bennett et al., 2009; Costa et al., 2016) encompasses 21 items organized into three domains: function (e.g. “difficulty in brush or comb hair,” “difficulty in preparing a homemade meal”), overall impact (e.g. “feeling completely overwhelmed by fibromyalgia symptoms”), and severity of symptoms (e.g. pain, sleep, fatigue). Participants rate each item using a 0 to 10 numeric scale in an increasing degree of severity and the past week as a timeframe. An overall score can be computed through the weighted sum of the three domains, with higher scores indicating greater fibromyalgia severity. The following cut-off points were considered to interpret this overall score: values >23 and ≤40 are mild; values > 40 and ≤ 63 are moderate; values between > 63 and ≤ 82 are severe; and values > 82 are very severe (Salaffi et al., 2021). In the present study, the main analyses only use the symptoms domain (*α* = .88). To avoid some degree of overlap and consequent overestimation of the model, the symptoms pertaining to psychological distress (depression and anxiety) were excluded from the symptom domain score. This change did not alter the adequacy of the subscale’s internal consistency (*α* =.82).

*The Hospital Anxiety and Depression Scale* (HADS; Pais-Ribeiro et al., 2007; Zigmond and Snaith, 1983) was applied. For this study, only the depression subscale was used (α = 0.84) to evaluate the severity of depressive symptoms in the past week. The HADS includes depression items rated on a 0 to 3 Likert scale. An example of an item is “*I feel as if I am slowed down.*” The interpretation of scores for depression is: 0–7 “normative”; 8-10 “mild”; 11–14 “moderate”; and 15–21 “severe” (Pais-Ribeiro et al., 2007). The original and the Portuguese versions presented good psychometric properties, including good internal consistency values and high temporal stability.

### Analytic strategy

Statistical procedures were performed with IBM SPSS software Statistics 25.0 (IBM Corp., 2017) and PROCESS macro, v3.5 (Hayes, 2017). Preliminary analyses were conducted to test the adequacy of data. Normality was ascertained by inspection of skewness and kurtosis values and through the Mardia’s test (Kim, 2013; Tabachnick and Fidell, 2012). Independence of residuals was tested via the Durbin-Watson test.

Descriptive statistics were performed to represent the sociodemographic and clinical characteristics of the sample and the variables under study. The presence, direction, and strength of association among variables were explored through Spearman rank-order correlations and interpreted using Cohen’s (1988) benchmarks: values ranging from 0.10 to 0.29 were considered weak, from 0.30 and 0.49 moderate, and above 0.50 large.

The demographic variables age and education years were not correlated with depressive symptoms and were, therefore, not included as covariates in analyses. A mediation model (Model 4) was conducted to examine the potential mediator role of shame in the relationship between fibromyalgia symptoms and depression. The potential moderator role of compassionate self-responding on the relationship between external shame and depressive symptoms was then tested by means of a moderation model (Model 1). Variables defining products, that is, external shame and self-compassion, were mean-centered for the analysis. The bootstrapping method, with 5000 resamples and a bias-corrected 95% confidence level (BCCI), was used to assess the direct and indirect associations of the variables (Hayes, 2017). Bootstrapping was chosen because it can deal with deviations from normal score distributions. A figure was used to interpret interactions (Aiken and West, 1991). Effects were considered statistically significant (at *p* < .05), when the 95% confidence interval did not enclose the value 0 (Hayes, 2017).

## Results

### Participants

The sociodemographic and clinical characteristics of the sample are presented in Table 1.

**Table 1.** Means (M), standard deviations (SD), frequencies (*n*), and percentages (%) of the sociodemographic and clinical variables (*N* = 138).

Variables	*M* ± SD
Age	47.9 ± 8.8
Education	13.7 ± 4.2
Age of onset of symptoms	32.0 ± 10.5
Age at diagnosis	40.5 ± 10.0
	*n* (%)
Marital status	
Single	16 (11.6%)
Married/co-habiting	101 (73.2%)
Divorced/separated	21(15.2%)
Working status	
Employed	92 (66.7%)
Unemployed	27 (19.6%)
Sick Leave	14 (10.1%)
Retired	5 (3.6%)
NUTS 2 regions	
Northern Portugal	33 (23.9%)
Central Portugal	52 (37.7%)
Lisbon Metropolitan Area	32 (23.2%)
Alentejo	9 (6.5%)
Algarve	7 (5.1%)
Autonomous Regions of the Azores and Madeira	5 (3.6%)
Who diagnosed	
Rheumatologist	103 (74.6%)
General Physician	9 (6.5%)
Other	26 (18.7%)
Comorbidities and treatment	
Medical comorbidities	69 (50.4%)
Psychiatric comorbidities	107 (78.1%)
Current use of medication	97 (70.8%)
Past or current psychological treatment	83 (60.6%)

*Note.* NUTS 2 = Nomenclature of Territorial Units for Statistics.

### Preliminary analyses

Analyses showed that the absolute and z-values of skewness and kurtosis were acceptable according to the recommended benchmarks (i.e. skewness < |3| and kurtosis < |8-10| and z-scores < 3.29; Kim, 2013; Tabachnick and Fidell, 2012) and did not show severe biases to univariate normality. Univariate skewness absolute and z-scores ranged between −0.03/−0.17 (for fibromyalgia severity) and 0.56/2.71 (for external shame), whereas univariate kurtosis ranged between 0.03/0.07 (for compassionate self-responding) and −0.58/−1.43 (for fibromyalgia severity). Multivariate normality was screened via Mardia’s test, with coefficients > |5| indicating non-normality (Byrne, 2010). Multivariate skewness and kurtosis were 2.03 and 33.82, respectively.

An inspection of the graphic representation of the variables showed four moderate outliers, which we opted to exclude from the data. No multicollinearity issues were detected, with VIF values ranging between 1.13 and 1.54, all below the reference guideline of VIF <5 (Kline, 2005). Independence of residuals was met with the Durbin-Watson test being within the recommended range (DW = 2). There were no missing data.

### Characterization of the variables

Means and standard deviations were computed for each variable and are displayed in Table 2. The mean overall FIQR score was 64.9 (SD = 16.2), reflecting a severe impact of FM. Results indicated that the mean FIQR score in the present study was close to that reported by prior studies (Bennett et al., 2009; Costa et al., 2016). Depressive symptoms, in turn, were mild on average, with 56 participants (40.6%) reporting normative scores, 41(29.7%) mild scores, 28 (20.3%) moderate scores, and 13 (9.4%) severe scores of depressive symptoms. Shame levels were greater than those found in the reference Portuguese population (Matos et al., 2015). Mean scores for self-compassion were low and similar to those reported previously for this condition (Carvalho et al., 2019; Sirois and Hirsch, 2019).

**Table 2.** Descriptive statistics (means, standard deviations, medians, and interquartile range) and Spearman’s Rank correlation coefficients (ρ) for the variables under study (*N* = 138).

					*ρ*		
Variable	*M* (SD)	Mdn (IQR)	1	2	3	4	5
1. Age	47.9 (8.8)	48.0 (43–55)	–	–	–	–	–
2. Education years	13.7 (4.2)	12.0 (12–17)	−.06	–	–	–	–
3. Fibromyalgia severity (FIQR-S)	28.0 (5.7)	28.0 (24–33)	.20*	−.37**	–	–	–
4. External shame (OAS2)	11.4 (7.3)	10.0 (6–16)	−.20*	−.19*	.26**	–	–
5. Compassionate self-responding (SCS)	3.1 (0.7)	3.1 (3–4)	.19*	.17	−.10	−.44**	–
6. Depressive symptoms (HADS-D)	8.6 (4.1)	9.0 (6–11)	−.02	−.14	.43**	.49**	−.41**

Note. FIQR-S: Revised-Fibromyalgia Impact Questionnaire –Symptoms Subscale; OAS2 = Other as Shamer Scale-2; SCS: Self-Compassion Scale; HADS-D: Hospital Anxiety and Depression Scale–Depression subscale.

*p ≤ 0.05. **p ≤ 0.01.

### Presence and magnitude of the associations

Spearman rank-order correlation coefficients are presented in Table 2. Statistically significant correlations were found between age and all variables, except for education years and depressive symptoms. These associations were positive and weak in magnitude. Education years showed significant negative correlations, ranging between weak and moderate, with external shame and fibromyalgia severity, respectively. Significant weak-to-moderate associations were found between fibromyalgia severity and external shame and depressive symptoms. External shame and compassionate self-responding were significantly associated with depressive symptoms, with similar magnitude but in opposite directions. Moreover, these variables were significantly, negatively, and moderately associated with each other.

### External shame as a potential mediator of the association between fibromyalgia severity and depressive symptoms

**Figure 1.**
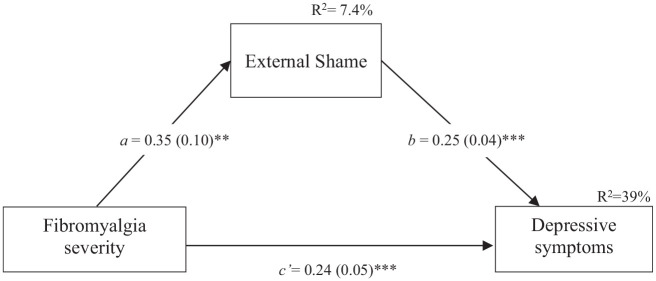
Statistical diagram of the mediation model (*N* = 138). Path values represent unstandardized coefficients. Numbers in parentheses represent standard errors. **p ≤ 0.01. ***p ≤ 0.001.

Figure 1 depicts the statistical diagram representing the tested mediation model. The simple regression model showed that fibromyalgia severity, alone, explained 21% of the variance of depression symptoms [*F*(1,136) = 35.33, *p* < 0.001). The addition of external shame to the model translated into an 18% increase in the amount of variation explained regarding depressive symptoms.

The estimated model was significant [*F*(2, 135) = 43.09, *p* < .001], accounting for 39% of depressive symptoms variance. The total effect, which comprises the sum of the direct effect (c’) and the indirect effect (a*b) was significant (Effect = 0.33, SE = 0.06, *p* < 0.001, CI = 0.22 to 0.43). Concerning main effects, in the final model both fibromyalgia severity (direct effect (*c’*) = 0.24, SE = 0.05, *p* < 0.001, 95%CI = 0.14 to 0.34) and external shame (*b* = 0.25, SE = 0.04, *p* < 0.001, 95%CI = 0.17, 0.33) were positively associated with depressive symptoms. These effects were additive, i.e., they remained significant after taking account of the other variables in the model. The indirect association of fibromyalgia severity on depressive symptoms through external shame was also significant (a*b = 0.09, SE = 0.03, BCCI = 0.03 to 0.15). This means that for people in which fibromyalgia severity is the same but external shame is different, those with more external shame are estimated to present 0.09 units higher depressive symptom levels.

### Compassionate self-responding as a potential moderator of the link between external shame and depressive symptoms

**Figure 2.**
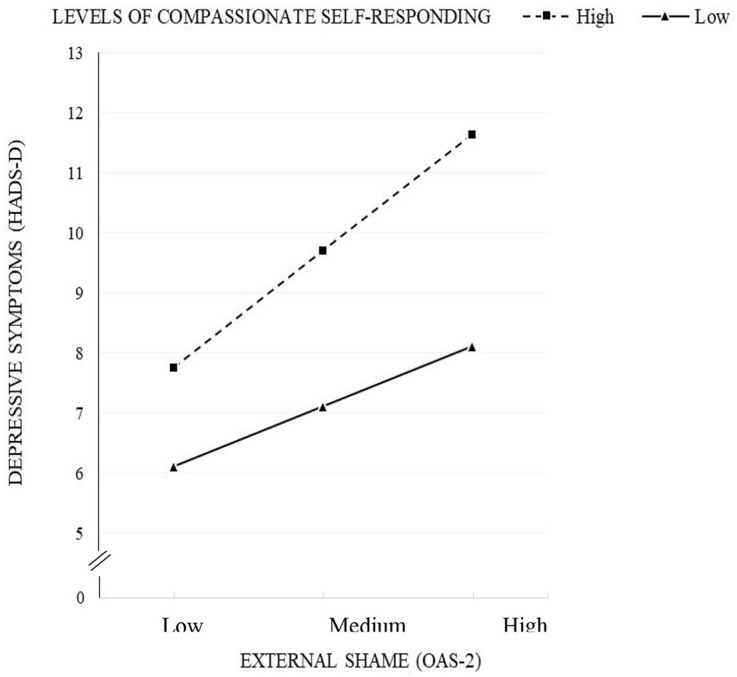
Depressive symptoms as a function of low (−1 SD) and high (+1 SD) external shame for low (−1 SD) and high (+1 SD) levels of compassionate self-responding (*N* = 138).

The estimated model was significant [*F*(3, 134) = 27.68, *p* < 0.001], accounting for 38% of depressive symptoms variance (Table 3). Both external shame and compassionate self-responding were additively and significantly associated with depressive symptoms. Over and above these main associations, the interaction of external shame and compassionate self was also associated with depressive symptoms: *b* = −0.09, SE = 0.04, *t* = −2.01, *p* = 0.047, 95% CI = [−0.173, −0.001]. This significant interaction indicates that the association between external shame and depressive symptoms varies as a function of levels of self-compassionate responding. Figure 2 shows this interaction: the association between external shame and depressive symptoms was weaker in people with a higher than lower compassionate self.

**Table 3.** Moderation of the relationship between external shame and depressive symptoms by self-compassionate responding (*N* = 138).

	*R* ^2^	MSE	*F* (df), *p*
Model	0.38	10.85	*F*(3,134) = 27.68, *p* < 0.001
	Coeff (SE)	*t, p*	95%CI
External shame (S)	0.20 (0.04)	4.54, *p* < 0.001	0.11 to 0.29
Compassionate self-responding (C)	−1.74 (0.43)	−4.05, *p* < 0.001	−2.60 to 0.89
S*C interaction	−0.09 (0.04)	−2.01, *p* = 0.047	−0.17 to −0.00

*Note*. Values represent unstandardized coefficients.

#### Ancillary analysis

We analyzed the models separately given the small sample size but did an ancillary moderated mediation analysis (Model 14) to get an indication of the complete model. While the mediation proved significant, results showed that the interaction term did not reach statistical significance (*p* = 0.053). In contrast, the index of moderated mediation was significant (Index = −0.0271; 95%bootCI = −0.0600 to −0.0006). Simple slope analysis showed that the slope for people low on self-compassion was significant (*b* = 0.22, *p* < 0.001), whereas the slope for people high on self-compassion was not significant (*b* = 0.10, *p* = 0.07). This analysis aligns with the results obtained in the separate models.

## Discussion

Understanding variability in individual factors that may promote or, in turn, buffer against depression is of utmost importance for an adequate assessment and treatment of fibromyalgia. This study sought to understand the potential relevance of external shame as a threat fostering depressive symptoms and the health-protective role of self-compassion in case of external shame. Findings supported our hypotheses that external shame may mediate the link between fibromyalgia severity and depressive symptoms and that the association between shame and depressive symptoms is weaker when compassionate self-responding is higher.

Correlation analyses revealed that fibromyalgia severity was positively associated with external shame and depressive symptoms and negatively with compassionate self-responding. This aligns with previous observations of relationships between these variables (Aguglia et al., 2011; Alciati et al., 2012; Marangell et al., 2011; Nordahl and Stiles, 2007).

The hypothesized mediation model was confirmed. The model suggests that the risk for depression is especially high if both the severity of symptoms and external shame are higher. Of note, it is impossible to do an unequivocal test of mediation using a cross-sectional design. However, whatever the precise role of external shame in an individual (cause, mediator, or consequence), its observed direct and indirect association with fibromyalgia and depressive symptoms indicate its potential relevance in fibromyalgia. Previous studies reported elevated shame levels among people with chronic musculoskeletal pain (Turner-Cobb et al., 2015). This observation and our current results suggest that management of external shame might be an option to reduce depression, especially in the case of severe fibromyalgia.

Self-compassion has been proposed to counteract the malignant impact of shame (Gilbert, 2010, 2017). We examined whether this could also be the case in fibromyalgia. Although associations between self-compassion and depressive symptoms uniformly suggest that compassionate self-responding may protect against depression (Carvalho, Trindade, et al., 2020; Muris and Petrocchi, 2017), this inference from a cross-sectional observation is only tentative. It is an inherent problem of self-report measures that they contain a substantial pervasive mood disposition of negative affectivity (Watson and Pennebaker, 1989). Moreover, other influences may cause this association, including the impact of depression on compassionate self-responding, influences of third factors such as attachment or childhood adversities (Gilbert, 2010; Mikulincer and Shaver, 2005; Pepping et al., 2015), as well as construct/item overlap and response tendencies (e.g. acquiescence, social desirability). Our results showed that, even when adjusting for external shame, compassionate self-responding was still associated with depressive symptoms. This additive (independent) association reflects that shame and compassionate self-responding have a different relation to depressive symptoms, highlighting the need to consider both factors when trying to understand or improve depressive symptoms.

Our study gave a stronger indication that compassionate self-responding might protect against depression in the moderator (interaction) analysis, because this analysis —at least partly— adjusts for covariances reflecting negative affectivity and response tendencies. Previous studies found a similar indication for a protective effect of compassionate self-responding (Carvalho et al., 2019; Carvalho, Trindade, et al., 2020). Longitudinal and clinical experimental research is needed to get a more thorough understanding of the directionality of associations as well as the changeability of variables.

Overall, these findings support the Fibromyalgia Imbalance of Threat and Soothing Systems (FITSS) model that postulates the importance of considering the balance between an overactive ’threat’ system and an underactive ’soothing’ system in fibromyalgia (Pinto, Geenen, et al., 2023). Fibromyalgia symptoms, shame, and depressive symptoms may contribute to the continuous activation of the threat-system, whereas self-compassion, as part of the soothing system, may dampen the nefarious consequences of such threat-related activation. Mindfulness and compassion-based approaches cultivate a warm, kind, and compassionate self-to-self-relationship, helping people with fibromyalgia develop a sense of safeness, regulating physiological reactivity, and reducing self-directed negative affectivity and perceived invalidation from others (Gilbert, 2005; Gooding et al., 2020; Maratos and Sheffield, 2020). Our study suggests that shame may also be countered by increasing compassionate self-responding.

Meta-analytic reviews with chronic physical illnesses and other clinical and non-clinical populations have shown that compassion-based approaches can improve pain-related outcomes, illness acceptance, isolation feelings, self-regulation skills (self-compassion, mindfulness), psychological distress, and well-being (Austin et al., 2021; Kılıç et al., 2021; Kirby et al., 2017). Regarding fibromyalgia, in particular, the few existing studies observed reductions in self-report measures of symptom severity, psychological distress as well as neurotrophins and pro-inflammatory markers along with increments in psychological flexibility, self-efficacy, and quality of life (Montero-Marin et al., 2017, 2019; Santos et al., 2022; Penlington, 2019). None of these studies included a measure of shame. Our study suggests that the assessment of shame in screening, monitoring, and evaluation of compassion-based approaches in fibromyalgia might be worthwhile. It would offer the possibility to examine in a longitudinal design whether shame is associated with intervention outcomes and whether changes in shame mediate the effect of therapy. The results of our study also call for health professionals to create a safe clinical environment—by embodying and modelling compassionate qualities—where patients can feel understood, accepted, and cared for (Gilbert, 2017).

Some caveats should be taken into consideration when interpreting these findings. The sample was recruited online and was entirely composed of women, which limits its representativeness and the generalization of results. There seem to be gender differences indicating higher severity and reporting of fibromyalgia and depressive symptoms (Wolfe et al., 2018; Yunus, 2002) and lower self-compassion levels (Yarnell et al., 2015) in women than men. Efforts to include men should be taken in future studies as a means to explore gender differences in the psychosocial makeup of patients and its association with different disease profiles, symptom-related trajectories, and outcomes. The study’s cross-sectional design is another limitation since it precludes drawing conclusions about causal relations among variables. Longitudinal and network studies are needed to better understand the dynamic interplay between these constructs over time. The incremental value of other constructs that may account for individual differences in shame-proneness and the risk of developing depression in people with fibromyalgia should also be addressed by future research (e.g. interpersonal adversity, body image concerns, self-criticism, fears of compassion). Research has also shown that shame characteristics differentially impact depression (e.g. depending on who the “shamer” is, the centrality and traumatic features of shame experiences), underscoring the need to consider this when assessing and working with shame (Matos, 2012). Although external shame has been more strongly linked to depression (Callow et al., 2021), it would also be important to assess the presence and relative contribution of internal shame in future studies.

In conclusion, our findings add to the current evidence by showing that external shame and compassionate self-responding may constitute relevant players in fibromyalgia and associated emotional distress. Both shame and self-compassion should be more widely investigated to understand and target the cognitive-affective influencing factors of fibromyalgia. This study also underscores the potential benefits of including self-compassion training in existing psychological interventions for the management of fibromyalgia.
